# Trapped by the Arrows: Avoidant/Restrictive Food Intake Disorder and the Illusion of Control in Type 1 Diabetes Mellitus

**DOI:** 10.7759/cureus.85539

**Published:** 2025-06-07

**Authors:** Maham Tahir, Adnan Zahid, Sara Afzal

**Affiliations:** 1 Internal Medicine, CMH Lahore Medical College and Institute of Dentistry, Lahore, PAK; 2 Endocrinology, Bahria International Hospital, Rawalpindi, PAK; 3 Internal Medicine, Bahria International Hospital, Rawalpindi, PAK

**Keywords:** avoidant restrictive food intake disorder (arfid), cgm, continuous glucose monitoring systems, diabetes mellitus management, disordered eating behavior, eating behaviors, flash glucose monitoring, picky eating behavior, type 1 diabetes mellitus (t1dm)

## Abstract

We present the case of a 25-year-old female patient with a 20-year history of type 1 diabetes mellitus (T1DM), who experienced recurrent episodes of shakiness, diaphoresis, and dizziness, despite normal blood glucose levels. These symptoms were attributed to changes in the direction of the arrows displayed on her continuous glucose monitoring (CGM) device. Further investigations, including capillary and venous blood glucose measurements, ruled out biochemical hypoglycemia. Psychiatric evaluation using the Diagnostic and Statistical Manual of Mental Disorders, Fifth Edition (DSM-5) criteria excluded anorexia nervosa, bulimia nervosa, diabulimia, generalized anxiety disorder (GAD), obsessive-compulsive disorder (OCD), and autism spectrum disorder (ASD). Upon analysis of her CGM data and clinical history, it became apparent that her restrictive eating behavior was driven by a fear of glycemic fluctuations, both hypoglycemia and hyperglycemia, rather than body image concerns. She was ultimately diagnosed with avoidant/restrictive food intake disorder (ARFID), primarily driven by worry about blood glucose instability. This case highlights the importance of distinguishing between psychological and physiological causes of glycemic fluctuations in T1DM and underscores the need for early intervention in patients with restrictive eating patterns.

## Introduction

Type 1 diabetes mellitus (T1DM) is a chronic autoimmune disorder characterized by insulin deficiency, requiring lifelong management with insulin therapy. Effective management of T1DM requires continuous monitoring and careful control of blood glucose levels to prevent both short-term (such as hypoglycemia) and long-term complications (including cardiovascular, renal, and retinal damage). However, many individuals with T1DM also experience significant psychological distress related to blood glucose management [1]. Fear of hypoglycemia (FoH) is a common phenomenon in T1DM, contributing to poor glycemic control and, at times, maladaptive behaviors such as insulin underuse or excessive correction of perceived lows [2,3]. Avoidant/restrictive food intake disorder (ARFID), a diagnosis in the Diagnostic and Statistical Manual of Mental Disorders, Fifth Edition, Text Revision (DSM-5-TR), involves restricted eating behaviors driven by the fear of adverse consequences of eating [4]. Although ARFID is commonly associated with a lack of interest in food, it can also manifest in individuals with diabetes who exhibit a fear of glycemic instability or complications, which leads to dietary restriction and an intense preoccupation with food-related decisions [5]. In this case report, we explore the complexities of managing a patient with T1DM who presented with symptoms suggestive of hypoglycemia, but whose underlying psychological distress was more consistent with ARFID than with traditional eating disorders such as anorexia nervosa or bulimia nervosa.

## Case presentation

A 25-year-old female patient with a 20-year history of T1DM, managed with insulin lispro before meals and insulin glargine at bedtime, and using continuous glucose monitoring (CGM) for the past eight years, presented with a two-year history of recurrent shakiness, diaphoresis, dizziness, and gradual weight loss. Each episode lasted approximately 10-15 minutes and occurred 3-4 times per week, with varying intensities ranging from mild discomfort to symptoms severe enough to prompt emergency department visits. Over 12 months, she lost approximately 6 kg, representing a 10%-12% reduction in body weight. Her BMI was 19 at presentation. These symptoms occurred despite her blood glucose readings being normal or elevated, both on her CGM and during evaluations in the emergency department. The patient reported experiencing these symptoms after noticing changes in the direction of the trend arrows on her CGM, particularly following food intake. Capillary blood glucose measurements were performed at multiple time points during her symptomatic episodes, and venous blood glucose samples were sent for laboratory confirmation. All values remained within normal or elevated ranges, effectively ruling out true biochemical hypoglycemia. She was not taking any medications other than insulin. The patient attributed the onset of symptoms to her increased engagement with diabetes-related literature, which heightened her awareness and fear of potential complications from both hypoglycemia and hyperglycemia.

**Figure 1:**
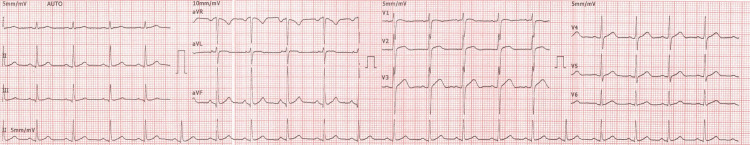
Normal ECG ECG: electrocardiogram.

On physical examination, her blood pressure was 120/80 mmHg while seated and 110/80 mmHg after standing for three minutes. While sitting comfortably, her pulse rate was 89 beats per minute, respiratory rate 18 breaths per minute, and oxygen saturation 98% on room air. Chest auscultation revealed clear and equal breath sounds bilaterally. Cardiovascular examination was unremarkable, with normal first and second heart sounds and no additional murmurs, gallops, or rubs. An electrocardiogram (ECG) was unremarkable (Figure 1). In the absence of palpitations, syncope, or other cardiovascular warning signs, cardiac arrhythmias were considered, but a Holter monitor was deemed unnecessary. The patient’s symptoms were temporally related to CGM feedback and food-related decision-making, suggesting a psychobehavioral component.

There were no signs of skin hyperpigmentation or palpable goiter on examination. Laboratory investigations, including thyroid function tests, morning cortisol, adrenocorticotropic hormone (ACTH), serum electrolytes, liver and renal function tests, troponin I, complete blood count, and anti-tissue transglutaminase immunoglobulin A (anti-tTG IgA), and total IgA, were all within normal limits (Table 1). The patient denied symptoms suggestive of gastroparesis, such as postprandial nausea or vomiting.

**Table 1:** The patient's laboratory test results HbA1c: hemoglobin A1c, TSH: thyroid-stimulating hormone, WBC: white blood cell, MCV: mean corpuscular volume, anti-tTG IgA: anti-tissue transglutaminase immunoglobulin A, IgA: immunoglobulin A, ACR: albumin-to-creatinine ratio, AST: aspartate aminotransferase, ALP: alkaline phosphatase, ACTH: adrenocorticotropic hormone.

Test	Patient value	Normal range
HbA1c	5.9%	Normal, A1c below 5.7%; prediabetes, A1c between 5.7% and 6.4%; diabetes, A1c of 6.5% or higher
TSH	2 mU/L	0.45-4.12 mU/L
WBC	5,000 cells/µL	4,000-11,000 cells/µL
Hemoglobin	12 g/dL	Male, 13-17 g/dL; female, 12-15 g/dL
Plasma free metanephrines	Plasma metanephrine, 0.2 nmol/L; plasma normetanephrine, 0.3 nmol/L	Plasma metanephrine, <0.5 nmol/L; plasma normetanephrine, <0.9 nmol/L
MCV	100 fL	80-100 fL
Anti-tTG IgA	1 U/mL	Negative, <4.0 U/mL; weak positive, 4.0-10.0 U/mL; positive, >10.0 U/mL
Total serum IgA	178 mg/dL	70-312 mg/dL
Serum sodium	137 mmol/L	135-145 mmol/L
Serum potassium	4 mmol/L	3.5-5.0 mmol/L
Serum calcium	9.5 mg/dL	8.5-10.2 mg/dL
Urine ACR	7.0 mg/g	<30 mg/g
Serum creatinine	1 mg/dL	0.6-1.2 mg/dL
AST	28 U/L	5-30 U/L
ALP	78 U/L	50-100 U/L
Vitamin B12	222 pg/mL	200-900 pg/mL
Serum folate	3.8 ng/mL	3-15 ng/mL
Serum ferritin	12 ng/mL	Male, 20-250 ng/mL; female, 10-120 ng/mL
Morning cortisol	20 µg/dL	6-23 µg/dL (8 AM)
ACTH	45 pg/mL	6-76 pg/mL

Neurological causes, including diabetic autonomic neuropathy, vestibular migraine, seizure disorders, and demyelinating disease, were considered. However, these were ruled out based on a normal neurological examination, absence of focal deficits, lack of postural or episodic symptom patterns, and the patient’s preserved awareness of glycemic fluctuations.

A comprehensive panel of laboratory and clinical investigations ruled out the medical causes of her symptoms. Notably, vitamin B12 levels were in the low-normal range (220 pg/mL), and serum folate was mildly reduced (3.8 ng/mL) (Table 1), indicating early nutritional compromise due to dietary restriction. These findings underscore the importance of early nutritional intervention in patients with restrictive eating behaviors. 

**Figure 2:**
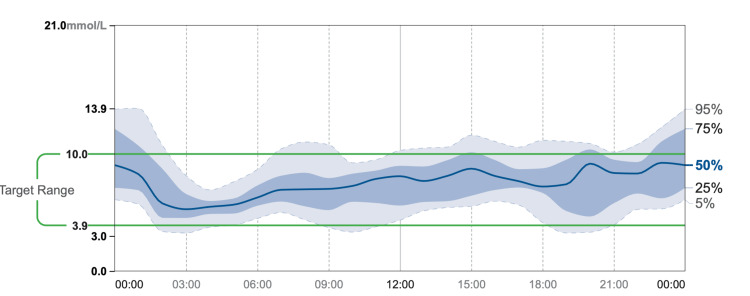
Time-based glucose percentile distribution from CGM over three months CGM: continuous glucose monitor.

**Figure 3:**
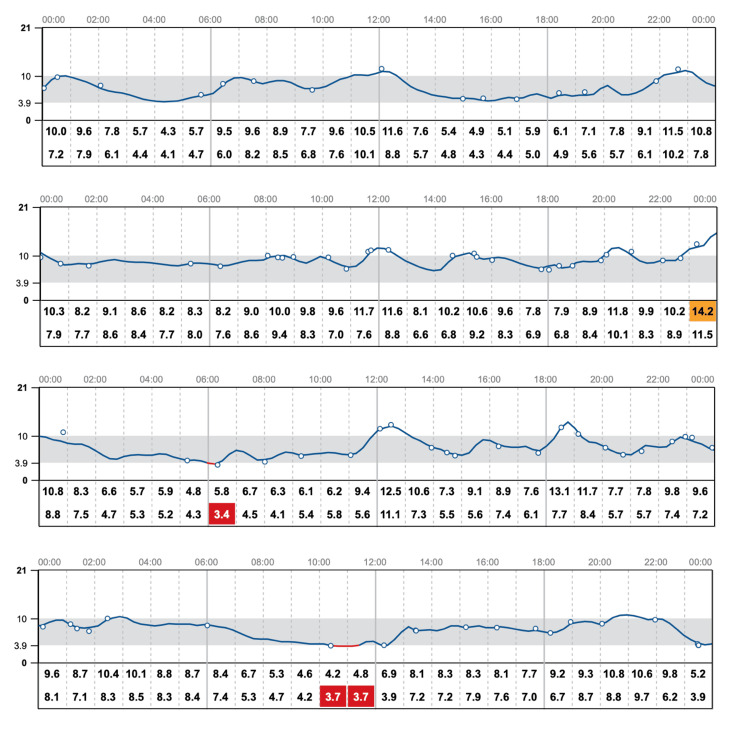
Daily CGM trend graphs CGM: continuous glucose monitor.

**Figure 4:**
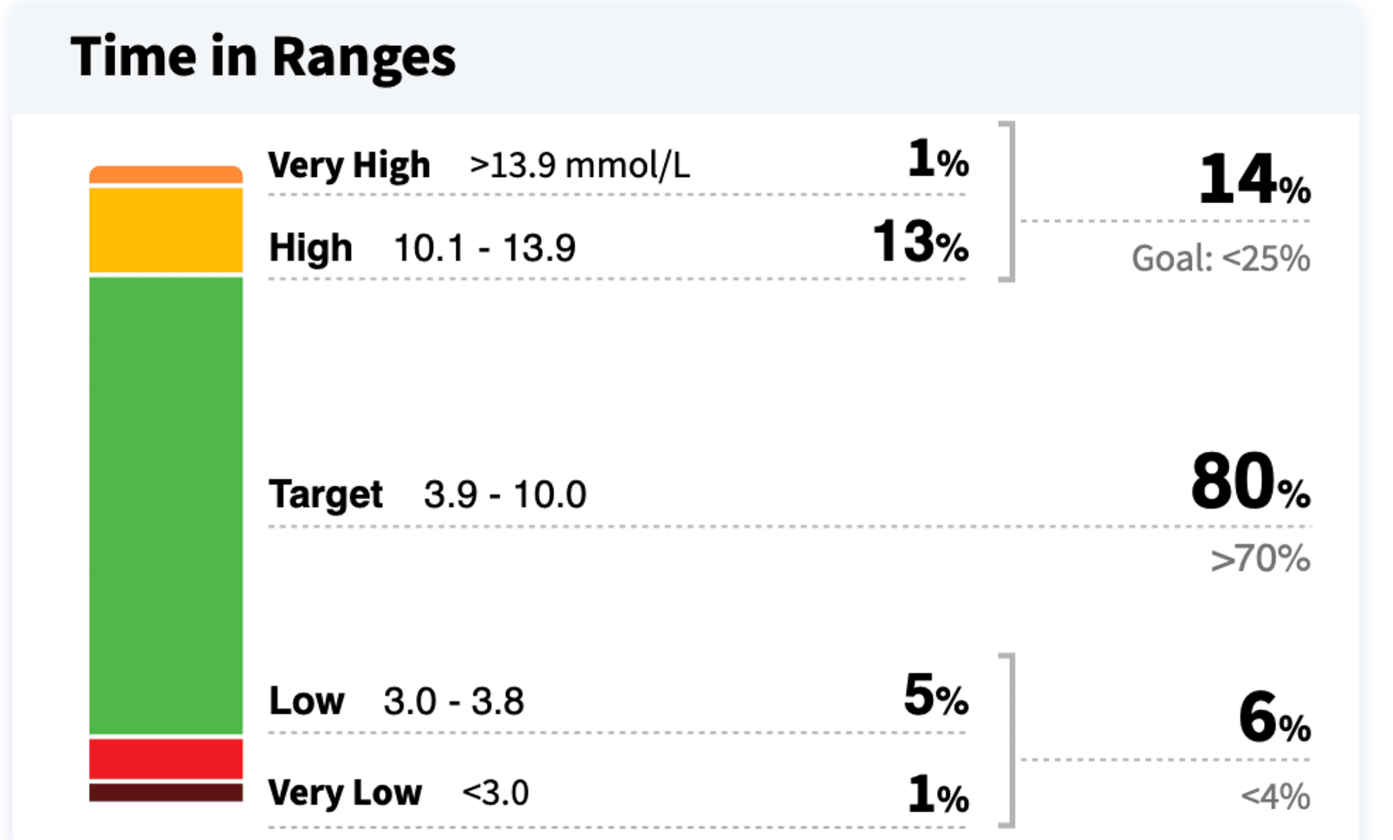
CGM summary: time in range, above, and below target levels over a one-month period CGM: continuous glucose monitor.

CGM data demonstrated relatively stable glucose trends over time, with limited variability. This stability was attributed to the patient’s avoidance of new foods and restricted meal intake due to concerns about their potential impact on blood glucose levels (Figures 2, 3). Additionally, CGM data showed that the patient’s glucose levels were within the target range 80% of the time, with 14% above and 6% below the target range (Figure 4).

These findings indicate overall good glycemic control. However, the patient's persistent fear of fluctuations, despite adequate time in range, suggests significant psychological distress related to glucose variability. Further exploration revealed that the patient consumed only one meal per day, supplemented by sugar-free beverages and green tea. She reported intentionally limiting food intake to avoid glycemic excursions that could disrupt the appearance of a “perfect” CGM graph (Figure 3). Additionally, she expressed anxiety about managing insulin doses for new or unmeasured foods and frequently avoided eating out due to fear of unpredictable postprandial glucose spikes.

Assessment by a psychiatrist using the DSM-5 criteria ruled out anorexia nervosa, bulimia nervosa, diabulimia, obsessive-compulsive disorder (OCD), generalized anxiety disorder (GAD), and autism spectrum disorder (ASD). The patient did not express any body image concerns or desire for weight loss. Instead, her eating behavior was driven by an intense fear of glycemic instability and diabetes-related complications. Based on these findings, she was diagnosed with ARFID, as defined by the DSM-5-TR criteria [6].

While the frequent overcorrection of perceived hypoglycemia might initially suggest characteristics of fear of hypoglycemia (FoH), the patient’s broader eating restriction, driven by both hypoglycemia and hyperglycemia concerns, along with her rigid control over food intake to avoid fluctuations in her glucose graph, aligns more closely with a diagnosis of ARFID, rather than isolated FoH.

The patient was educated about the acute complications of hypoglycemia, including its potentially life-threatening nature, as well as the long-term complications of sustained hyperglycemia. Strong emphasis was placed on the accurate interpretation of CGM data, particularly the significance of trend arrows and the importance of avoiding unnecessary overcorrection. Cognitive Behavioral Therapy (CBT) was initiated to address anxiety and rigid thought patterns related to glycemic control and food intake. Family counseling was also conducted to support collaborative blood glucose management and reduce diabetes-related stress within the household. 

A structured meal plan was developed in consultation with a registered dietitian to ensure adequate energy intake, balanced macronutrient distribution, and gradual desensitization to unfamiliar or feared foods. Nutritional guidance included increasing the intake of folate-rich foods such as leafy greens (e.g., spinach, kale), legumes (e.g., beans, lentils), citrus fruits, and fortified cereals and bread, in response to low-normal folate levels. To address her fear of consuming new or unmeasured foods, the patient was also booked for a carbohydrate counting course, aimed at building confidence in insulin dosing and improving dietary flexibility. Follow-up with an endocrinologist was advised to reassess insulin needs and monitor progress in the context of the revised dietary and behavioral management strategies.

## Discussion

The patient, a 25-year-old female with a 20-year history of T1DM, presented with recurrent episodes of shakiness, diaphoresis, and dizziness, which she associated with hypoglycemia despite normal or elevated blood glucose levels. These episodes, combined with a markedly restricted eating pattern and an overreliance on CGM feedback, suggested a psychobehavioral component rather than a physiological cause for her symptoms.

In patients with T1DM, the FoH is a common psychological phenomenon where individuals overcorrect perceived low blood sugar levels, resulting in hyperglycemia and further anxiety. FoH is linked to both behavioral and cognitive patterns, such as excessive carbohydrate intake, insulin underuse, or eating more frequently to prevent lows [2,3]. The interplay between eating disorders and diabetes forms a complex cycle, where each condition can significantly affect the development and progression of the other, leading to intricate physiological, psychological, and behavioral interconnections [7].

The patient restricts her intake to one meal per day and avoids eating additional food out of intense anxiety about blood glucose fluctuations and maintaining a stable CGM graph. This eating pattern is not motivated by body image concerns but by fear of the aversive consequences of eating. The behavior has resulted in inadequate nutritional intake and marked interference with psychosocial functioning. While occurring in the context of diabetes, the severity and rigidity of the restriction exceed what is typically expected in diabetes self-management and meet diagnostic criteria for ARFID [8].

Concerns about long-term complications are a frequent psychological burden for individuals managing diabetes, affecting those with both type 1 and type 2 forms of the condition. This apprehension can influence emotional well-being and contribute to suboptimal health outcomes. It is often intertwined with heightened diabetes-related stress and may be accompanied by signs of depression [9].

Laboratory investigations revealed early nutritional compromise, with low-normal vitamin B12 and mildly reduced folate levels, suggesting that dietary restrictions were impacting her nutritional status. This is consistent with the finding that ARFID can result in nutritional deficiencies [10]. ARFID involves a pattern of severely limited eating that can result in weight loss, inadequate nutrition, or reliance on supplements. Unlike anorexia nervosa or bulimia nervosa, this condition is not driven by concerns about body shape or weight [4].

In managing this case, a dual focus on medical stabilization and behavioral modification was essential. The patient’s misinterpretation of CGM trend data, particularly the direction of trend arrows, highlighted a significant gap in health literacy, contributing not only to inappropriate carbohydrate correction but also to a fear of food intake, which contributed to restrictive food intake [11]. A meta-analysis examining the role of CBT in eating disorders concluded that CBT is an effective treatment approach for this population [12]. Recognizing the psychological challenges faced by patients with diabetes enables healthcare providers to implement tailored strategies that support better disease management and lower the impact of complications [13]. Effective diabetes management requires the integration of individual, social, and family support systems. Strengthening family bonds, in particular, plays a crucial role in fostering healthy self-care behaviors and overcoming barriers to maintaining stable blood glucose levels [14]. Learning carbohydrate counting gives individuals with diabetes greater autonomy in planning meals, snacks, and daily activities while supporting balanced nutritional habits. This method may contribute to improved blood sugar regulation, a lower risk of hypoglycemic episodes, and enhanced overall well-being without significantly affecting body weight [15,16]. The American Dietetic Association emphasizes that nutrition intervention, particularly individualized counseling provided by a registered dietitian (RD), is a vital part of comprehensive care for individuals with eating disorders. This support should be integrated throughout both the assessment and treatment phases across all levels of care [17]. A cross-sectional study found that adolescent girls with type 1 diabetes are nearly twice as likely to experience DSM-IV-defined and subclinical eating disorders compared to their peers without diabetes, highlighting the need for further investigation in this area [18].

Managing patients with disordered eating is most effective when approached through a multidisciplinary team model. This typically involves collaboration among a physician, a registered dietitian, and a mental health specialist, each bringing their own expertise to support the patient [19]. A recent systematic review and meta-analysis reported that automated insulin delivery (AID) systems are associated with decreased diabetes management burden and improved psychological well-being in individuals with diabetes [20]. However, AID was not a feasible therapeutic option in this case due to limited availability and healthcare resource constraints in the country where this case was managed.

## Conclusions

This case highlights a unique presentation of ARFID in a patient with T1DM, where the psychological distress surrounding blood glucose fluctuations led to restrictive eating patterns. While the patient's symptoms might initially suggest FoH, her broader avoidance of food, driven by both hypoglycemia and hyperglycemia concerns, aligns more closely with ARFID. This underscores the importance of distinguishing between psychological conditions such as ARFID and other eating disorders in patients with diabetes. Early recognition of ARFID, alongside appropriate psychological and nutritional interventions, is crucial to preventing further health complications and improving the patient’s quality of life.
